# MicroRNA-145 Targets *YES* and *STAT1* in Colon Cancer Cells

**DOI:** 10.1371/journal.pone.0008836

**Published:** 2010-01-21

**Authors:** Lea H. Gregersen, Anders B. Jacobsen, Lisa B. Frankel, Jiayu Wen, Anders Krogh, Anders H. Lund

**Affiliations:** 1 Biotech Research and Innovation Centre and Centre for Epigenetics, University of Copenhagen, Copenhagen, Denmark; 2 The Bioinformatics Centre, Department of Biology, University of Copenhagen, Copenhagen, Denmark; University of Edinburgh, United Kingdom

## Abstract

**Background:**

MicroRNAs (miRNAs) have emerged as important gene regulators and are recognized as key players in tumorigenesis. miR-145 is reported to be down-regulated in several cancers, but knowledge of its targets in colon cancer remains limited.

**Methodology/Principal Findings:**

To investigate the role of miR-145 in colon cancer, we have employed a microarray based approach to identify miR-145 targets. Based on seed site enrichment analyses and unbiased word analyses, we found a significant enrichment of miRNA binding sites in the 3′-untranslated regions (UTRs) of transcripts down-regulated upon miRNA overexpression. Gene Ontology analysis showed an overrepresentation of genes involved in cell death, cellular growth and proliferation, cell cycle, gene expression and cancer. A number of the identified miRNA targets have previously been implicated in cancer, including *YES*, *FSCN1*, *ADAM17*, *BIRC2*, *VANGL1* as well as the transcription factor *STAT1*. Both *YES* and *STAT1* were verified as direct miR-145 targets.

**Conclusions/Significance:**

The study identifies and validates new cancer-relevant direct targets of miR-145 in colon cancer cells and hereby adds important mechanistic understanding of the tumor-suppressive functions of miR-145.

## Introduction

In past years small non-coding RNAs have been recognized as important gene regulators [1]–[4]. miRNAs are an abundant group of endogenous small non-coding RNAs that function as regulators of protein encoding genes through translational repression and/or degradation of their target mRNAs [1]. Extensive miRNA research has revealed that miRNAs are involved in the regulation of numerous key cellular functions such as metabolism, cell proliferation, tumorigenesis, apoptosis, development and differentiation [1], [3], [4]. To regulate target mRNAs mature miRNAs are bound to AGO proteins and guide the AGO-associated RNA induced silencing complex (RISC) to mRNA targets through imperfect base pairing between the miRNA and the target. This often involves perfect base paring between the 5′ end of the miRNA strand and its target, also termed the seed site. Bioinformatics analyses suggest that each miRNA can control hundreds of target genes in humans and it has recently been reported that over 60% of protein encoding genes are under selective pressure to maintain pairing with miRNAs, indicating that miRNAs have the potential to regulate the majority of protein encoding genes [5].

Inappropriate expression of miRNAs, which regulate genes functioning as either tumor-suppressors or oncogenes can ultimately lead to acquisition of the hallmarks of cancer, thus specifying miRNAs as both tumor-suppressors and oncogenes [3], [6], [7]. Specific changes in miRNA expression levels have been associated with various types of cancer [8] and a large number of miRNAs are localized in so-called cancer-associated genomic regions, which are frequently exposed to changes in cancer cells [9]. However, in contrast to the large number of miRNAs that has been identified in the past years, only relatively few miRNA targets have been experimentally validated. Given the overwhelming evidence that miRNAs are important regulators of tumorigenesis [3], [6], identification of miRNA targets is necessary in order to understand the mechanistic basis for the involvement of miRNAs in cancer.

miR-145 has frequently been reported as down-regulated in cancers, including prostate cancer [10], [11], bladder cancer [12], colon cancer [13]-[18], ovarian cancer [19], [20] as well as B-cell malignancies [21], [22], and it has been reported that the genomic region encoding miR-145 is located in a fragile site often deleted in cancer [23]. Accordingly, miR-145 overexpression has been demonstrated to have a growth inhibitory effect [16], [17], [24], [25] and to suppress anchorage independent growth [16]. It has furthermore been demonstrated that miR-145 expression is induced by p53 [16]. Here, it was reported that miR-145 targets c-Myc through imperfect seed base pairing [16] and it was suggested that p53-mediated downregulation of c-Myc is, at least partially, due the p53-mediated upregulation of miR-145. Another study also found an increased level of miR-145 induced by doxorubicin [26]. However, instead of a transcriptional activation of miR-145 it was found that p53 activates the processing of primary miR-145 transcripts into miR-145 precursors [26]. This phenomenon was not only restricted to miR-145, but also applied to several other miRNAs with known growth suppressive functions, indicating p53-mediated regulation of miRNA processing as a way of exerting its tumor-suppressive function [26]. In addition to c-Myc, miR-145 has also been suggested to target the human insulin receptor substrate-1 (IRS-1) and type I insulin-like growth factor receptor (IGF-IR) in colon cancer [25], [27]. However, the specificity of the target interaction was not confirmed by mutational analysis of the seed sites in either of these cases.

The expression level of miR-145 is induced during differentiation of human embryonic stem cells and miR-145 overexpression has been demonstrated to impair the pluripotency in human embryonic stem cells by targeting of *OCT4*, *SOX2* and *KLF4* that are involved in maintaining the self-renewing capacity of human embryonic stem cells [28]. This role of miR-145 as an inducer of differentiation in embryonic stem cells is in agreement with the role of miR-145 as a repressor of growth in cancer cells. A mouse model designed to investigate the expression pattern of miR-145 revealed expression of miR-145 in multipotent cardiac progenitors and smooth muscle cells [29] and suggested that miR-145 promotes the differentiation into smooth muscle cells [29]. However, another study showed that mice lacking miR-145 were viable with no overt abnormalities in smooth muscle cell differentiation [30], demonstrating the existence of additional promoters of differentiation.

Taken together, miR-145 appears to have tumor-suppressor functions when overexpressed in cancer cells and may normally play a role in differentiation processes. Here, we have focused on target identification of miR-145 in colon cancer, based on microarray expression profiles of cells overexpressing miR-145. Gene Ontology analyses of miR-145 responsive genes confirm regulation of genes involved in cell death, cell cycle regulation and cancer. We have identified a number of miR-145 targets that could be interesting in a cancer context and verified that miR-145 targets YES and STAT1 in colon cancer cells.

## Results

**Figure 1:**
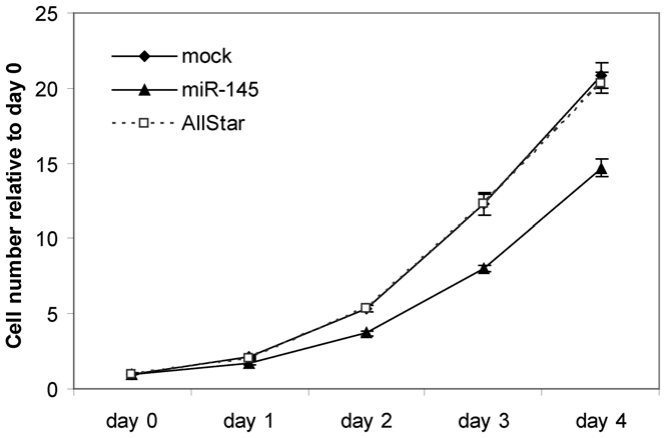
miR-145 overexpression reduces the proliferative potential of DLD-1 cells. DLD-1 cells transfected with 50 nM miR-145 duplex exhibit a reduced cell proliferation as measured by crystal violet growth assay. Data are shown as the mean ± S.D. of four replicates.

Prompted by the numerous reports of miR-145 downregulation in cancer we sought to identify miR-145 targets that could explain the role of miR-145 in cancer. To gain insight into the expression levels of miR-145 in established cell lines, we profiled the expression levels in a number of cancer cell lines as well as in non-tumorigenic cell lines (FIGURE S1). The expression profiles of miR-145 showed that except for MCF10A all tested non-tumorigenic cells lines expressed miR-145, whereas the expression levels of miR-145 were very low in all tested cancer cell lines, supporting previous findings where miR-145 is down-regulated or lost in cancer. Due to its implications in colon cancer [13]–[18], we decided to investigate the role of miR-145 in the colon cancer cell line DLD-1. Co-transfection of miR-145 duplex with a luciferase reporter containing a perfect complementary site to the mature miRNAs resulted in a marked downregulation of the luciferase activity, demonstrating a highly effective overexpression (FIGURE S2A). As demonstrated by both crystal violet and MTT growth assays, overexpression of miR-145 resulted in a decreased cell proliferation (FIGURE 1 and FIGURE S3).

### Identification of miR-145 Targets

Given the indications that miR-145 plays a role in cancer, together with the limited knowledge of its targets in colon cancer, the aim was to identify functionally relevant targets that could help to explain the role of miR-145 in cancer. To achieve this, we used a microarray based strategy similar to the one previously used to identify miR-21 targets [31]. DLD-1 cells were transfected with 50 nM miR-145 duplex or mock transfected. Total RNA was harvested 24 hours post-transfection and analyzed on Affymetrix HG-U133 Plus 2.0 human arrays. A total of eight arrays were analyzed. For filtering, uninformative genes with the same expression level across all arrays (including non-expressed genes) were removed and the differentially expressed genes, their corresponding p-values and false discovery rates were calculated as described in the MATERIALS AND METHODS section.

To determine if the genes regulated upon miR-145 overexpression were related to specific cellular functions, a search within the functional annotations in the Ingenuity database (Ingenuity® Systems, www.ingenuity.com) was performed for all miR-145 responsive genes. The top five enriched functional categories of the Gene Ontology analysis are listed in TABLE 1. Among these categories are cell death, cellular growth and proliferation, cell cycle regulation and cancer. Corresponding analyses using random gene sets generated from Affymetrix HG-U133 Plus 2.0 annotations, in some cases resulted in enrichment of the same categories that we found enriched in the miR-145 dataset, but with p-values 10 orders of magnitude higher than in the miR-145 dataset (data not shown).

**Table 1:** Enriched functional categories among miR-145 responsive genes.

Function	P-values*	Number of Molecules
Cell Death	3.36·10^−23^–7.86·10^−4^	514
Cellular Growth and Proliferation	1.13·10^−21^–6.93·10^−4^	531
Cell Cycle	3.08·10^−20^–7.99·10^−4^	273
Gene Expression	6.16·10^−18^–7.99·10^−4^	361
Cancer	9.23·10^−17^–8.09·10^−4^	571

*P-values indicating enrichment of functional subgroups within the indicated functional categories.

**Figure 2:**
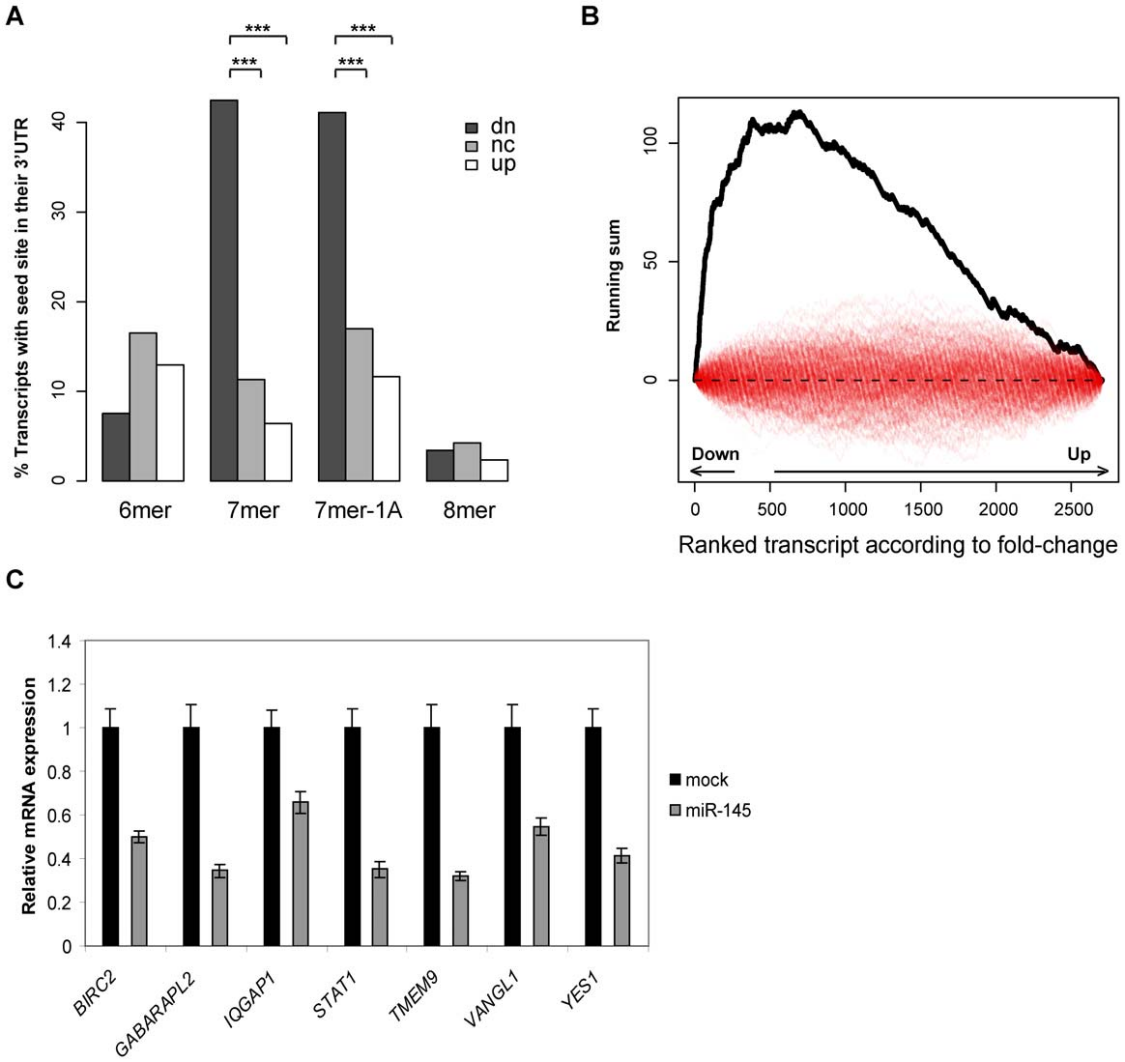
Microarray based identification of miR-145 targets. **A**, The percentages of genes in the up, down (dn) and no-change (nc) sets with seeds sites in their 3′UTRs. Only those 6mers which are not part of a 7mer or 8mer are reported as 6mers and only those 7mers that are not contained within a 8mer are counted as 7mers. Mean log fold-changes were 0.36, 0.02 and −0.40 for the up, down and no-change sets, respectively. The p-values are calculated testing the null hypothesis that the proportion of genes with seed sites are the same for the down-regulated and the up-regulated genes (dn vs. up) or the down-regulated genes compared to the no-change genes (dn vs. nc). P-values for 7mer seed site enrichment were 1.7·10^−33^ (dn vs. up) and 2.9·10^−11^ (dn vs. nc). P-values for 7mer-1A seed site enrichment were 3.7·10^−18^ (dn vs. up) and 7.8·10^−7^ (dn vs. nc). **B**, Unbiased word analysis showing the running sum of the overrepresentation score for the miR-145 7mer seed site in the ranked list of 3′UTR sequences (black line) compared to permutations of the ranked gene list (red lines). Down- and up-regulated genes (defined as genes with FC<−1.1 or FC>1.1) in the ranked gene list are indicated in the graph. **C**, Quantitative RT-PCR validation of the microarray data. Cells were transfected with 50 nM miR-145 duplex or mock transfected and total RNA harvested 24 hours post-transfection. The 3′UTRs of *IQGAP1* and *STAT1* do not contain any miR-145 seed matches, but both genes contain a 7mer seed match in their coding region. All other miR-145 responsive genes contain at least one 7mer seed site in their 3′UTR. The expression level of each transcript is shown relative to the level in mock transfected cells. Data are shown as the mean ± S.D. of three replicates.

Seed site enrichment analysis of the occurrences of miR-145 seed sites in the 3′UTRs demonstrated a highly significant enrichment among the down-regulated transcripts (FIGURE 2A). The p-values for the enrichment of miR-145 seed sites (including 7mer, 7mer-1A and 8mer sites) were 1.4·10^−21^ and 7.6·10^−8^ when considering the down-regulated transcripts vs. up-regulated transcripts and down-regulated transcripts vs. no change transcripts, respectively. Two alternative methods of calculating the seed site enrichment, either as seed site occurrences after correcting the up, down and no changes sets to the same size or as seed site occurrences per kb, showed a similar enrichment in the down-regulated 3′UTRs (FIGURE S4). Taken together, this verifies that the microarray based approach can be used to identify miRNA targets. When the whole cDNA sequence of the transcripts was used for seed site enrichment analysis (reported as the percentage of transcripts with seed sites), we still find a significant enrichment of seed sites, even though the enrichment is less pronounced compared to only considering the 3′UTR sequences (FIGURE S5A). A similar analysis of coding sequences and 5′UTR sequences revealed no significant seed site enrichment (FIGURE S5B and S5C).

Unbiased word analyses of the 3′UTR sequences identified the 7mer seed site of miR-145 as the most significantly enriched 7mer word in the 3′UTRs of down-regulated transcripts (TABLE 2). Several of the other highly enriched words were variations of the miR-145 seed site and the fourth most enriched word was the 7mer-1A seed match (TABLE 2). A corresponding 6mer word analysis also identified miR-145 seed matches as the most significantly enriched words (FDR<0.005) (FIGURE S6). Plotting the running sum of the overrepresentation scores of the 7mer seed site in transcripts ranked according to their logFC clearly showed that the 3′UTRs of down-regulated transcripts had a high enrichment of miR-145 7mer seeds (black line) which was not observed for permutations of the ranked gene list (red lines) (FIGURE 2B).

**Table 2:** Enriched 7mer words in the 3′UTRs of down-regulated transcripts.

Rank	Word*	z-Score	FDR	Annotation
1	**AACTGGA**	17.21	<0.002	hsa-miR-145
2	CAGGAAA	16.76	<0.002	
3	CCAGGAA	13.94	<0.002	
4	**ACTGGA**A	13.88	<0.002	
5	T**ACTGGA**	8.54	<0.002	
6	A**AACTGG**	8.49	<0.002	
7	AATCCCA	8.19	<0.002	
8	**ACTGGA**T	7.91	<0.002	
9	TCAGGAA	7.85	<0.002	
10	T**AACTGG**	7.62	<0.002	

*Underlined sequences correspond to complete or partial miR-145 seed site match.

### Potential miR-145 Targets

Since miR-145 is down-regulated or lost in cancer, re-introduction of miR-145 would be expected to cause a direct downregulation of oncogene targets. Indeed, a number of genes with oncogenic function were down-regulated upon miR-145 overexpression including the Src family member *YES* and the actin cross-linking protein *FSCN1*, which is found in cell surface projections implicated in cell motility [32]. The metalloproteinase *ADAM17* and a member of the inhibitor of apoptosis (IAP) family *BIRC2* (also known as *cIAP1*), which both contain 8mer miR-145 seed sites in their 3′UTRs, were also observed as down-regulated upon miR-145 overexpression. In addition, *VANGL1* which is involved in wound healing response of intestinal epithelial cell lines through promotion of cell migration was also identified as a putative miR-145 target [33].

Some of the down-regulated genes did not have a miR-145 seed site in their 3′UTR, but instead had one or more a seed sites in the coding region. STAT1, a well known transcription factor [34], [35] and IQGAP1 a negative regulator of cell-cell adhesions and stimulator of cell motility and invasion [36], both had miR-145 seed sites in the coding regions of their transcripts. In the case of *STAT1*, the miR-145 seed site is located in the second last exon of the transcript. Since *STAT1* was among the most down-regulated transcripts in the microarray analysis, it was also considered as a potential target and included in the subsequent investigations. The previously identified miR-145 targets *OCT4*, *SOX2* and *KLF4* in human embryonic stem cells were not expressed in DLD-1 cells (data not shown). *MYC*, *ISR-1* and *IGF-1R*, suggested by others as miR-145 targets [16], [25], [27], showed no change in expression level upon miR-145 overexpression in our dataset (data not shown). The full list of identified potential miR-145 targets containing seed sites in their 3′UTR is presented in TABLE S1.

### Target Validation

**Figure 3:**
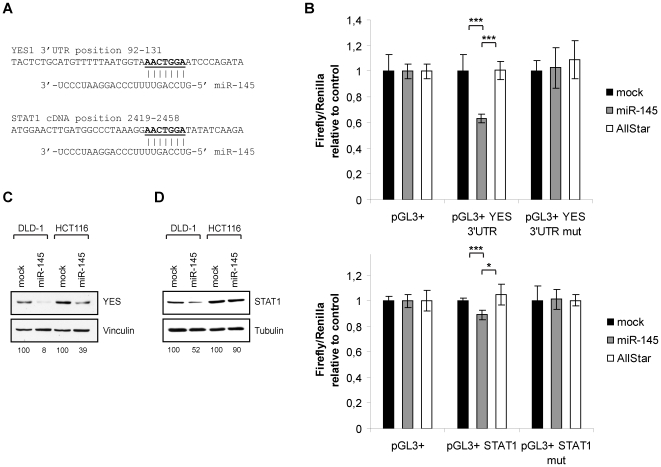
Targets validation of YES and STAT1. **A**, Sequence alignment of the miR-145 seed region and mRNA targets. The position coordinates are indicated for the transcript isoforms listed below. *YES1* 3′UTR: ENSG00000176105:ENST0000035983 *STAT1* cDNA: ENSG00000115415:ENST00000361099. **B**, Firefly luciferase assay with pGL3+ constructs holding a 3′UTR fragment of *YES* or a cDNA fragment of *STAT1*, downstream to the firefly luciferase gene. Cells were co-transfected with firefly luciferase reporters along with a *Renilla* luciferase transfection control plasmid either alone or with 50 nM miR-145 duplex and 50 nM AllStar duplex as a negative control. In pGL3+ mut vectors two base pairs in the miR-145 seed site have been mutated. Luminescence was measured 24 hours post-transfection and the ratio of the firefly to the *Renilla* activity is shown relative to transfection without RNA duplex and the empty vector. Data are shown as the mean ± S.D. of four replicates. *, p<0.05 using a two-tailed t-test, ***, p<0.01 using a two-tailed t-test. **C** and **D**, Western blot analysis of DLD-1 and HCT116 cells transfected with miR-145 duplexes or mock transfected cells blotted for YES (**C**) and STAT1 (**D**). Vinculin or tubulin were used as loading controls. The bands were quantified relative to the appropriate loading controls using the TotalLab software and are shown relative to the protein level in mock transfected cells. The data shown are representative of two experiments.

We next confirmed the microarray data by quantitative RT-PCR for seven selected transcripts that were found down-regulated in the microarray analysis (FIGURE 2C). Notably, *STAT1* and *YES*, which have miR-145 seed sites in the coding region and 3′UTR, respectively, showed a clear down-regulation on the transcript level upon miR-145 overexpression. To determine whether miR-145 directly regulates *YES* and *STAT1*, luciferase reporter constructs were cloned. The base pairing between the miRNA seed sites and the potential mRNA targets are depicted in FIGURE 3A. In both cases, overexpression of miR-145 resulted in decreased luciferase activity, indicating that the miRNA can bind both the *YES* 3′UTR and the *STAT1* cDNA and directly mediate repression (FIGURE 3B). This was not the case with reporter constructs where the seed sites had been mutated (FIGURE 3B). To further confirm the miRNA mediated down-regulation of STAT1 and YES on protein level, western blot analyses were performed in both DLD-1 cells and HCT116 colon cancer cells. The overexpression efficiency of miR-145 in HCT116 measured by luciferase reporter constructs was similar to that observed in DLD-1 cells (FIGURE S2B). A marked decrease of YES protein level mediated by miR-145 was observed 48 hours post-transfection in both DLD-1 and HCT116 cells (FIGURE 3C). A more subtle, but still clear downregulation of STAT1 mediated by miR-145 was also observed (FIGURE 3D). Notably, the degree of STAT1 repression varied between DLD-1 and HCT116 cells, with the strongest repression of STAT1 observed in DLD-1 cells.

### Secondary Effects Due to STAT1 Deregulation

STAT1 is a well characterized transcription factor best recognized as a transcriptional activator, but examples of negative transcriptional regulation have also been described [34]. The STAT1 binding motif as defined by the JASPAR CORE database [37] is shown in FIGURE S7A. To determine if the miR-145 mediated downregulation of STAT1 also caused a change of the expression level of STAT1 target genes in the microarray analysis, a search for STAT1 binding sites in the promoters of the down-regulated, up-regulated and transcripts with no change in expression level was conducted. Out of all JASPAR core transcription factors, STAT1 was the most significantly enriched binding site both when the down-regulated (p-value = 4.18·10^−4^) and the up-regulated genes (p-value = 1.34·10^−4^) were compared to the genes with no change in expression (TABLE 3). This was not the case for a permuted STAT1 binding matrix when comparing up-regulated genes to the genes with no change in expression level (FIGURE S7B). However, when considering the down-regulated genes compared to no-change genes, there was also a slight enrichment of STAT1 permuted binding sites, indicating that some of the observed effects may be non-specific (FIGURE S7C).

**Table 3:** Top 10 enriched or depleted transcription factor binding sites in the promoters of miR-145 responsive genes.

Down-regulated set vs. no-change set	Percentage	Percentage	P-value	Up-regulated set vs. no-change set	Percentage	Percentage	P-value
	Down	No-change			Up	No-change	
**STAT1**	70%	51%	4.18·10^−4^	**STAT1**	66%	51%	1.34·10^−4^
**FOXF2**	86%	73%	3.86·10^−3^	**IRF2**	27%	36%	1.73·10^−2^
**NFKB1**	71%	84%	4.84·10^−3^	**FOXF2**	8%	73%	3.90·10^−2^
**ATHB5**	99%	91%	5.30·10^−3^	**Lhx3**	83%	77%	5.16·10^−2^
**Lhx3**	89%	77%	5.44·10^−3^	**HMG-IY**	99%	96%	6.76·10^−2^
**Pax5**	45%	59%	1.37·10^−2^	**Staf**	44%	51%	7.42·10^−2^
**NF-kappaB**	94%	99%	3.42·10^−2^	**RELA**	93%	96%	1.13·10^−1^
**Staf**	39%	51%	3.73·10^−2^	**HNF1A**	69%	63%	1.17·10^−1^
**FOXD1**	100%	96%	5.65·10^−2^	**Pax5**	65%	59%	1.19·10^−1^

## Discussion

miRNAs are emerging as important gene regulators and intensive research of their functions and targets have revealed a role of miRNAs in several key cellular functions. Even though our understanding of functional roles of miRNAs in cancers is steadily increasing, knowledge about miR-145 and its targets in colon cancer is still largely missing. We hence focused on the identification of miR-145 targets in colon cancer cells. Supporting previous findings that miR-145 is down-regulated in tumors [10]–[13], [15], [16], [18]–[21], [38]–[42], a profile of miR-145 expression in established cell lines demonstrated that expression levels of miR-145 were drastically reduced in cancer cell lines relative to non-tumorigenic cell lines. In agreement with reports showing a growth inhibitory effect of miR-145 [16], [17], [41], we also observe a growth reduction of DLD-1 cells upon transient miR-145 transfection, which implies that miR-145 possesses a tumor-suppressor function *in vitro*.

Here, we used microarray profiling upon miR-145 overexpression as a method of identification of potential targets. Microarray expression profiles combined with seed site enrichment analysis is a well established approach used to identify potential miRNA targets [31], [43], [44]. It has the advantage over strictly computational target prediction that it is not only based on the presence of a seed site and sequence features of the potential target, but takes into account whether the target is expressed in the considered cell line and whether the target is regulated on the transcript level. Since this approach is solely based on changes observed on transcript level, targets exclusively regulated by translational repression will not be identified.

Seed site enrichment analysis and unbiased word analysis showed a clear enrichment of miR-145 seed sites in the 3′UTRs of down-regulated transcripts, confirming our approach to identify miR-145 targets. We did not observe any enrichment of miR-145 seed sites in the coding region of down-regulated transcripts. However, since some of the genes containing seed sites within the coding region previously had been implicated in cancer, we speculated that these target candidates may also play a functional role downstream miR-145. The unbiased word analysis, used here to evaluate the enrichment of words in 3′UTRs according to the logFC of the transcript upon miR-145 overexpression, presents an extremely useful method for visualization of the effects of miRNA overexpression without making any prejudiced assumption regarding cutoff values. The shape of the running sum curve shows a sharp peak of the miR-145 7mer seed sites for the most down-regulated genes suggesting that target transcript down-regulation to a large part is due to direct effects of miR-145 targeting. The unbiased word analysis confirmed previous reports showing that an A at position 1 of the seed site is beneficial regardless of its potential to base pair with the miRNA [2], [45].

Many of the targets identified here have previously been implicated in cancer. The enriched functional categories of all miRNA responsive targets further supported the implication of miR-145 in cancer and imply that both the direct effects of miR-145 as well as secondary effects can explain the role of miR-145 in cancer. A number of genes with oncogenic function, many of which have also been associated with colon cancer, were identified as potential miR-145 targets based on the microarray analysis. These include *ADAM17*, *BIRC2* and *VANGL1*. ADAM17 has been reported up-regulated in colon carcinomas and has been shown to promote the release of epidermal growth factor receptor (EGFR) ligands from the cell membrane, thus activating EGFR [46]. BIRC2 has been demonstrated to be essential for maintaining endothelial cell survival and vascular integrity in zebrafish by activating the formation of the TNF receptor complex I as well as promoting the degradation of IκB, upon which NF-κB is released and translocates into the nucleus where it activates pro-survival genes in endothelial cells [47]. The cell membrane associated fraction of VANGL1 increases with differentiation and was demonstrated to co-localize with E-cadherin in human colon cells [33]. In addition, it was demonstrated that overexpression of *VANGL1* stimulated wound closure of intestinal epithelial cell lines, whereas siRNA directed against Vangl1 inhibited the migratory response [33]. Previously reported miR-145 targets including *OCT4*, *SOX2* and *KLF4* involved in the promotion of stem cell proliferation were not expressed in DLD-1 cells, implying that the growth inhibitory effect of miR-145 overexpression in DLD-1 cells is not due to downregulation of these genes. Another key regulator of cell proliferation, *MYC*, previously reported as a miR-145 target showed no change in expression level upon miR-145 overexpression in our setting.

Experimental validation of *YES* and *STAT1* as miR-145 targets demonstrated a marked down-regulated on mRNA and protein level, proving the biological effect of miR-145 on the endogenous targets. The down-regulation in 3′UTR/cDNA luciferase assays further validates that this is the result of direct interactions, since mutations of the miR-145 seed sites abolish this down-regulation. The reason of the different effects of miR-145 in the different assays is likely due to a lack of direct comparability between these assays. This difference could also be due to other binding factors involved in regulation of the endogenous transcript, as these binding sites are not present in the 3′UTR or cDNA fragments used in the cloned luciferase reporter constructs.

A number of studies have linked increased expression of YES in cancer with increased cell motility and tumor invasion [48], [49]. YES is part of the Scr kinase family [50] and its tyrosine kinase activity has been shown to be elevated in colonic adenomas compared to its activity in adjacent normal mucosa [51]. Furthermore YES activity was found to correlate with the predicted cancer risk based on size, histology, and degree of dysplasia [51]. Taken together, the present data suggests that upregulation of YES is important for growth and transformation of intestinal cells.

STAT proteins function downstream of JAKs and MAPKs, which induce the dimerization of STAT proteins, thereby allowing the translocation of STAT proteins into the nucleus [34], [35]. STAT1 is best known for its pro-apoptotic role in response to interferons, but STAT1 has also been reported to have a pro-survival role in some cancers [35]. We observed a downregulation of *STAT1* on the transcript level and protein level upon miR-145 overexpression. Furthermore, we show that this downregulation of STAT1 translates into an effect on the expression level of potential STAT1 targets. This is the case for genes both positively and negatively regulated by STAT1, but the effect is the greatest in genes negatively regulated by STAT1. Even though STAT1 has been reported both as a transcriptional activator and repressor, the main mechanism of STAT1 transcriptional regulation is the activation of its target genes [34]. Our findings suggest that transcriptional repression is more widespread than recognized previously. To sum up, transcription factor analyses of the promoters of deregulated genes provides a means to characterize secondary effects as previously published for miR-34a [52]. The identified enrichment of STAT1 binding sites in promoters of regulated genes demonstrates that secondary effects of miRNA overexpression are pronounced already 24 hours post-transfection. Hence, it is important to consider secondary effects when analyzing miRNA overexpression datasets.

In conclusion, using a microarray based approach we have identified additional targets for the cancer-associated miRNA miR-145 in colon cancer cells. The miRNA targets identified in this study may serve to clarify the role of miR-145 in colon cancers as well as other cancer types.

## Materials and Methods

### Cell Cultures

DLD-1 cells were cultured in DMEM GlutaMAX™-I high glucose medium (31966, Gibco Invitrogen) supplemented with 10% (v/v) fetal bovine serum (FBS, CH30160.03, Hyclone), 50 U/ml penicillin and 50 µg/ml streptomycin (P/S, 15140 Gibco Invitrogen) and incubated in 5% CO_2_ plus 20% oxygen. HCT116 cells were cultured in McCoy's 5A L-Glutamine medium (22330, Gibco Invitrogen) supplemented with 10% (v/v) FBS and P/S. Overexpression of miR-145 was achieved by transfection with a miR-145 duplex that mimics the mature miR-145 (PM11480; Ambion). Transfection with *Caenorhabditis elegans* lin-4 duplex (PM10768, Ambion) or AllStar negative control siRNA (1027281, Qiagen) was used as control. All transfections were carried out using Lipofectamine™ 2000 Transfection Reagent (11668-019, Invitrogen) according to the manufactures protocol. Unless otherwise stated cells were transfected with 50 nM of oligonucleotides. The transfection efficiency was evaluated by transfection with the modified, fluorescent RNA duplex siGLO Green Transfection Indicator (D-001630-01-20, Dharmacon).

### Quantitative RT-PCR

Total RNA was isolated with TRIZOL (15596-026, Invitrogen). RNA samples for mRNA quantitative reverse transcription PCR (RT-PCR) were treated with DNaseI (DNase-free kit™, Ambion, AM1906). Quantitative PCR primers were designed using the QuantPrime software [53] and sequences are listed in TABLE S2. The levels of mRNAs were quantified using the comparative CT method relative to levels of hypoxanthine phosphoribosyltransfease (*HPRT*). Mature miR-145 levels were quantified using TaqMan® MicroRNA Assay (4373133, Applied Biosystems) Quantification was normalized to the U6 small nuclear B non-coding RNA (RNU6B) which served as an endogenous control (4373381, Applied Biosystems).

### Cell Proliferation Assay

Cells were transfected with miR-145 duplex, AllStar negative control or mock transfected as described above and seeded in 24-well plates in four replicates the following day. For the crystal violet growth curves, cells were washed twice in PBS and fixed by addition of 1 ml 10% formalin to each well for 10 min., washed twice in water and air-dried. The fixed cells were stained with 0.1% crystal violet solution by shaking incubation for 30 min. Excess of crystal violet straining was removed by several washes with water. The plates were allowed to dry and crystal violet was extracted by addition of 250 µl to 1 ml of 10% acetic acid to each well depending on the amount of crystal violet staining followed by shaking incubation for 30 min. The amount of crystal violet staining was quantified by measurement of the absorbance at 570 nm. The day after the cells were seeded in 24-well plates were set to day 0 and used as a reference for the following days. For the MTT assay cells were incubated with 0.5mg/ml MTT for four hours. Following incubation the media removed and formazan extracted by 90% isopropanol and 10% formic acid solution. Absorbance was measured at 560 nm and reference wavelength at 750 nm was subtracted.

### Microarray Profiles

DLD-1 cells were transfected with miR-145 duplex or mock transfected in four biological replicates. Total RNA was isolated with TRIZOL 24 hours after transfection. Affymetrix microarray analysis (HG-U133 Plus 2.0 human) was performed at the Microarray Centre, Rigshospitalet, Copenhagen University Hospital as previously described [31]. Data processing and word analysis are described in separate sections below.

### Vector Construction and Reporter Assays or Plasmid Vectors

The miR-145 pMIR-REPORT luciferase reporter vector was cloned by inserting a oligo with perfect complementarity to mature miR-145 into the HindII/SpeI site of pMIR-REPORT Luciferase vector (AM5795, Applied Biosystems). Antisense and sense oligonucleotide sequences (with restriction overhangs) are as follow:

miR-145 AS: 5′-CTAGTAGGGATTCCTGGGAAAACTGGACGCTCAGCA-3′,

miR-145 S: 5′-AGCTTGCTGAGCGTCCAGTTTTCCCAGGAATCCCTA-3′,

The antisense and sense oligonucleotides were annealed in 50 µl reactions containing 20 µM oligos in annealing buffer (30 mM HEPES-KOH pH 7.4, 100 mM KCl, 2 mM MgCl_2_, 50 mM NH_4_Ac) prior to restriction and ligation into the pMIR-REPORT vector.

3′ UTR fragment of YES was PCR amplified from DLD-1 genomic DNA and cloned into the pGL3 control vector with a multiple cloning site inserted downstream of the luciferase gene (here called pGL3+) described previously [31]. An approximate 800bp fragment of *STAT1* cDNA containing miR-145 seed site were cloned from total DLD-1 cDNA. The primer sequences used for PCR amplification were as follows (restriction sites indicated in lower case):

*YES* 3′UTR BglII FW: 5′- GGGagatctATGCACAAATCTGCCAAAAT-3′

*YES* 3′UTR XhoI RV: 5′- GGGctcgagCATTTCCCCTTTGATTGGAC -3′

*STAT1* cDNA BglII FW: 5′- GGGagatctTGGGCTCAGCTTTCAGAAGT-3′

*STAT1* cDNA XhoI RV: 5′- GGGctcgagAGGAAAACTGTCGCCAGAGA-3′

The seed sites of miR-145 were mutated by substitution of two nucleotides in the seed site using the QuikChange site-directed mutagenesis kit (Stratagene) following the instructions of the manufacturer. The miR-145 7mer AACTGGA was converted into AACTCCA in the STAT1 vector and AAGAGGA in the YES vector. Mutagenesis primers used were as follows:

*STAT1* mut FW:5′-CTTGATGGCCCTAAAGGAACTCCATATATCAAGACTGAGTTGAT-3′

*STAT1* mut RV: 5′-ATCAACTCAGTCTTGATATATGGAGTTCCTTTAGGGCCATCAAG-3′

*YES* mut FW:5′-TCTTCTTTACTCTGCATGTTTTTAATGGTAAAGAGGAATCCCAGATATGGT -3′

*YES* mut RV: 5′-ACCATATCTGGGATTCCTCTTTACCATTAAAAACATGCAGAGTAAAGAAGA -3′

### Luciferase Assays

Cells in 96-well plates were transfected with 100 ng/well pMIR-REPORT and 25/well ng pRL-TK (E2241, Promega) together with increasing amount of miRNA duplexes. Firefly and *Renilla* luminescence was measured 24 hours after transfection using the Dual-Glo luciferase kit (E2940, Promega). Non-transfected cells were used for background subtraction and the ratio of luminescence from the firefly reporter relative to the luminescence from the *Renilla* control reporter was calculated. For 3′UTR luciferase validation of miR-145 targets DLD-1 cells/well were transfected with 75ng/well pGL3+ firefly luciferase reporter constructs together with 25 ng/well pRL-TK vector alone or with miR-145 duplex or AllStar negative control.

### Antibodies and Western Blot Analysis

For western blotting DLD-1 or HCT-116 cells were transfected twice on two subsequent days. Cells were harvested 48 hours after the first transfection, washed twice in PBS, and lysed in RIPA buffer (150 mM NaCl, 1% NP40, 0.5% sodium deoxycholate, 0.1% SDS, 50 MTris-HCl at pH 8, 2 mM EDTA) containing protease inhibitor cocktail (04693124001, Roche)) and phosphatase inhibitors (1mM NaVO_3_, 10mM NaF and 1mM β-glycerolphosphat). 30µg protein/lane was separated on polyacrylamide gels, transferred to a nitrocellulose membrane and incubated with antibodies against YES (2734, Cell Signaling) or STAT1 (9171, Cell Signaling) or antibodies against Tubulin (ab11304, Abcam) or Vinculin (V9131, Sigma) as a loading control. Band intensities were quantified using TotalLab image analysis software.

### Data Processing of Microarray Profiles

The expression data was processed using the “affy” package in BioConductor in R [54]. Probe set intensities were summarized using the Robust Multichip Average method and then transformed to generalized log values with the variance stable VSN method [55]. Due to different overall log2 intensity distribution and problems with fitting of the microarray, the fourth mock replicate array was removed prior to further analysis (FIGURE S8). The probe sets for the remaining arrays were subsequently mapped to Ensembl transcripts using the mappings provided by BioMart. Probe sets that mapped to more than two different Ensembl genes were discarded. For genes with more than one probeset mapped to it, the probeset with the largest inter quartile range of expression intensity was selected. Non-specific filtering was used to remove genes with low variance between arrays using a cutoff of 0.25. This left 2963 genes that were used for the following analysis. Differentially expressed genes were found using limma [56]. The p-values were corrected for multiple testing using the Benjamini and Hochberg's method to control the false discovery rate and genes were divided into up, down and no-change sets. Histograms of the logFC distributions before and after non-specific filtering are shown in FIGURE S8. As seen from FIGURE S8F the logFC distribution was displaced towards the up-regulated genes with a mean logFC of 0.226 after non-specific filtering. The reason for this displacement is not known, but it is most likely due to a technical artefact since the displacement is present in the logFC distributions before non-specific filtering. Genes with a FDR<0.25 were taken as either up- or down-regulated based on their log2 fold-change (logFC). A third set of genes that do not change from control to experiment was defined by selecting genes with FDR>0.8. The microarray data is MIAME compliant and have been deposited in the GEO database under the accession number: GSE18625.

### Seed Site Enrichment

For the analysis of seed site enrichment, miRNA sequences from miRBase (Release 12.0: Sept 2008) were used. 3′UTR sequences were retrieved using BioMart. For 3′UTRs with more than one transcript isoform the longest isoform was used. The 3′UTRs of the transcripts were scanned for matching 6mers, 7mers, 7mer-1As and 8mers (perfect 8-nucleotide match). In the definition used, seed sites are not contained in each other. This means that a 7mer, 7mer-1A or 8mer seed sites do not count as 6mer sites, corresponding to the definition used by TargetScan (http://www.targetscan.org/). In addition to the seed site enrichment reported as percents of transcripts in each set with seed matches, the seed site enrichment was also calculated as simple seed site counts after correcting the up, down and no-change sets to have the same size (scaling down the sets to the size of the smallest). In order to check that the seed site enrichment is not due to difference in 3′UTR length in the up, down and no-change sets, the seed site enrichment analysis was also performed where the seed site occurrences were counted per kb. The difference in the number of seed sites in transcripts from the up, down, and no-change sets was evaluated testing the null hypothesis that the proportions (probabilities of success) in the up, down, and no-change set are the same. Enriched functions and diseases associated with genes with a logFC above 1.1 or below -1.1 were found using the Ingenuity Pathways Analysis™ (IPA) release 8.0 (Ingenuity® Systems, www.ingenuity.com).

### Unbiased Word Analysis

We used a non-parametric statistical framework for scoring and ranking oligonucleotide words based on their overrepresentation in a ranked list of sequences. Our implementation closely follows a method for inferring miRNA activities in gene expression data that has previously been described by Cheng and Li [57]. We modified the original method by Cheng and Li in three areas. 1) While Cheng and Li only assess miRNA target sites, our implementation evaluates all words of a given length. 2) Cheng and Li aggregate miRanda target site scores per 3′UTR as a continuous measure for miRNA targeting, but because we evaluate all words we compute an overrepresentation score of a given word in 3′UTR sequence. For a given sequence and word, we estimate a p-value for the null hypothesis that the number of observed word occurrences *m* can be explained by the length and nucleotide composition of the sequence. The null distribution of word occurrences *P(m)* is estimated from 5000 mononucleotide permutations of the original sequence. The p-value, converted into a log-odds score, can be directly estimated by: 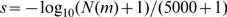, where *N(m)* is the number of shuffles where the word occurred *m* times or more. We add 1 as a crude correction for sampling uncertainty and to avoid log(0). 3) Finally, we calculated the running sum directly from overrepresentation scores (without weighing by absolute expression changes). This simpler statistic was found to work well in on public domain data sets (data not shown). Similar to Cheng and Li, we obtained a false discovery rate for each word by comparing word z-scores with z-scores calculated from all permutations of the ranked list of 3′UTRs.

### Motif Search for Overrepresented Transcription Factor Binding Sites

The promoter regions (2000bp upstream to the transcription start site) of the up, down and no-change sets were retrieved using BioMart and used to search for transcription factor binding sites as defined in the JASPAR CORE database [37]. All 123 core transcription factor binding count matrices were downloaded from the JASPAR website (http://jaspar.cgb.ki.se/). The count matrices were log-transformed into position specific weight matrices (PSSMs) using a background nucleotide frequency of 25% and pseudocount of 1. The search for matches to these PSSMs in the promoter sequences were performed with the Asap software package [58] using a threshold of 0.8. The percentage of promoters with hits for a given transcription binding site was compared between the down and the no-change sets or between the up and no-change sets. The p-values were calculated testing the null-hypothesis that the proportion of promoters with hits for a given transcription factor was the same in the two compared gene sets.

## Supporting Information

Figure S1 miR-145 expression profile. Endogenous expression levels of miR-145 determined by miRNA quantitative RT-PCR. The expression levels are shown relative to the non-coding RNA U6 which serves as an endogenous control. Data are shown as the mean ± S.D. of three replicates.
(0.01 MB PDF)

Figure S2 Validation of miR-145 overexpression. Firefly luciferase reporter containing a miR-145 (pMIR-145-REPORT) complementary site (perfect antisense sequence) was co-transfected with a Renilla luciferase transfection control plasmid and the indicated amounts of miRNA duplexes in DLD-1 cells (A) and HCT-116 cells (B). Transfection with lin-4 was used as a non-specific control. Luminescence was measured 24 hours post-transfection and the firefly luciferase activity was normalized to the activity of the co-transfected Renilla plasmid. Data are shown as the mean ± S.D. of four replicates.
(0.01 MB PDF)

Figure S3 MTT cell proliferation assay. DLD-1 cell proliferation as measured by MTT assay upon transfection with 50 nM miR-145 duplex, 50 nM AllStar negative control or mock transfection. Data are shown as the mean ± S.D. of four replicates.
(0.01 MB PDF)

Figure S4 Seed site enrichment reported per kb and as counts. A, Seed site occurrences in the 3′UTRs of up, down and no-change transcripts for miR-145 presented per kb. The p-values were calculated as described in FIGURE 2. P-values for 7mer seed site enrichment were 2.3.10-28 (dn vs. up) and 5.1.10-7 (dn vs. nc). P-values for 7mer-1A seed site enrichment were 1.3.10-14 (dn vs. up) and 8.7.10-4 (dn vs. nc). B, Seed site occurrences in the 3′UTRs of up, down and no-change transcripts for miR-145 after correction of the up, down and no-change gene sets to the same size (scaling down the sets to the size of the smallest). The p-values were calculated as described in FIGURE 2. P-values for 7mer seed site enrichment were 3.7.10-13 (dn vs. up) and 1.7.10-8 (dn vs. nc). P-values for 7mer-1A seed site enrichment were 1.4.10-7 (dn vs. up) and 1.2.10-5 (dn vs. nc).
(0.01 MB PDF)

Figure S5 Seed site enrichment reported for cDNA sequences, coding regions and 5′UTRs. The percentage of genes in the up, down and no-change sets with seeds sites calculated for the entire cDNA sequences (A), coding regions (B) and 5′UTRs (C). P-values for 7mer seed site enrichment in cDNA sequences were 7.7.10-10 (dn vs. up) and 1.3.10-3 (dn vs. nc). P-values for 7mer-1A seed site enrichment were 6.7.10-6 (dn vs. up) and 1.5.10-3 (dn vs. nc).
(0.01 MB PDF)

Figure S6 6mer unbiased word analysis. A, Running sum of the overrepresentation score for the miR-145 6mer seed site in the ranked list of 3′UTR sequences (black line) compared to permutations of the ranked gene list (red lines). B, Top 10 enriched 6mer words in the 3′UTRs among the down-regulated transcripts.
(0.41 MB PDF)

Figure S7 Enrichment of putative STAT1 binding sites in the promoters of up- and down-regulated genes. Binding motif for STAT1 as defined in the JASPAR database (A). Overrepresentation of STAT1 binding sites compared to a shuffled STAT1 binding site in promoters of down-regulated (B) and up-regulated (C) genes compared to promoters of genes with no change in expression level.
(0.10 MB PDF)

Figure S8 Microarray quality and processing. Histogram of raw log intensities for individual arrays before normalization (A). Boxplot of log intensities after normalization (B). The fit of the vsnrma model used to normalize the arrays were evaluated by calculation of normalized unscaled standard errors (C) together with relative log expression (RLE) plots showing the log expression for each probeset on each chip, relative to the median value for that probeset (D). Histogram of the log fold-change (logFC) distribution before (E) and after (F) non-specific filtering.
(0.06 MB PDF)

Table S1 List of potential miR-145 targets (defined as downregulated transcripts with at least one miR-145 7mer, 7mer-1A or 8mer seed site in their 3′UTRs).
(0.11 MB DOC)

Table S2 Primer sequences used for quantitative RT-PCR.
(0.03 MB DOC)
