# Supplementary figures and images for: MicroRNA-145 Targets YES and STAT1 in Colon Cancer Cells
Source: PLoS One. 2010 Jan 21;5(1):e8836. doi: 10.1371/journal.pone.0008836 (PMC2809101; doi:10.1371/journal.pone.0008836)

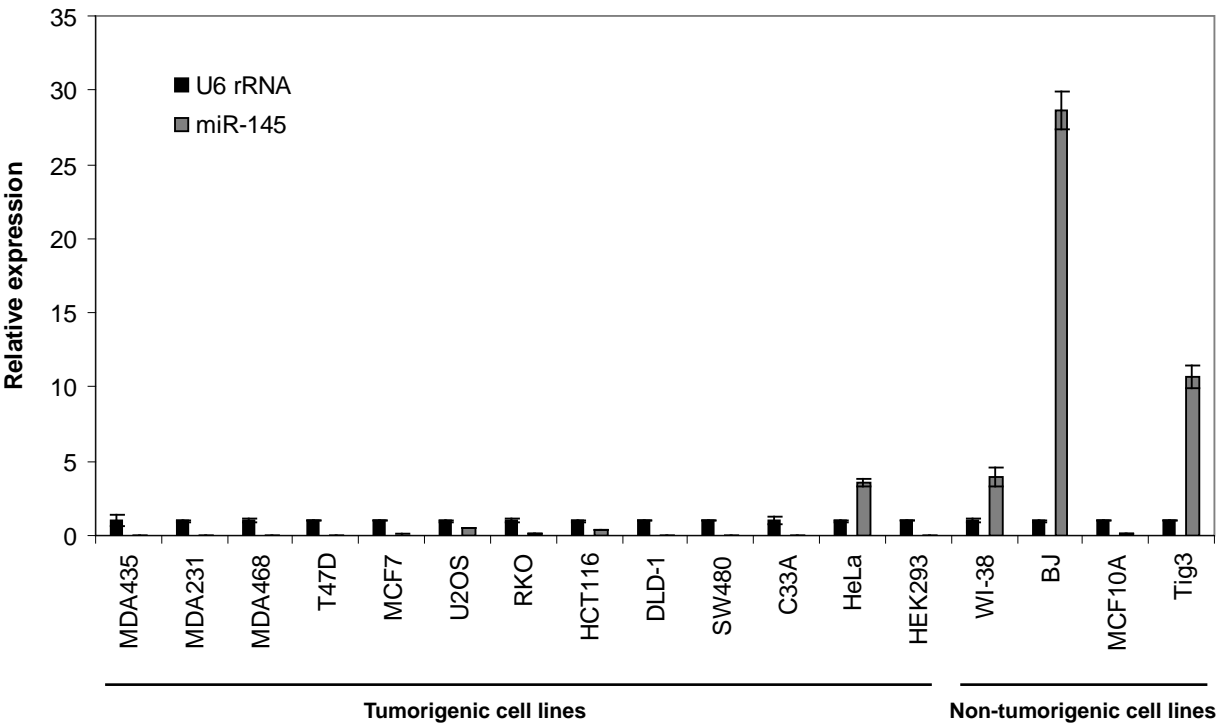

Supplement: Figure S1 — miR-145 expression profile. Endogenous expression levels of miR-145 determined by miRNA quantitative RT-PCR. The expression levels are shown relative to the non-coding RNA U6 which serves as an endogenous control. Data are shown as the mean ± S.D. of three replicates. (0.01 MB PDF) [file pone.0008836.s001.pdf]

**A**

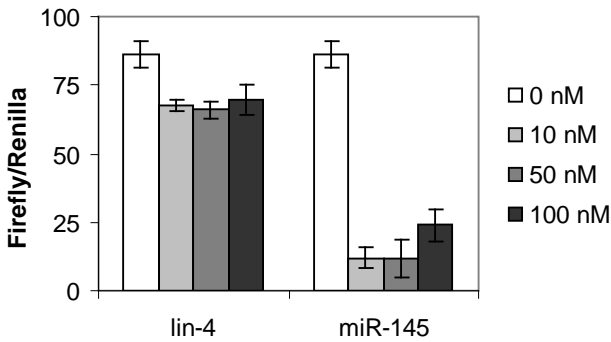

**B**

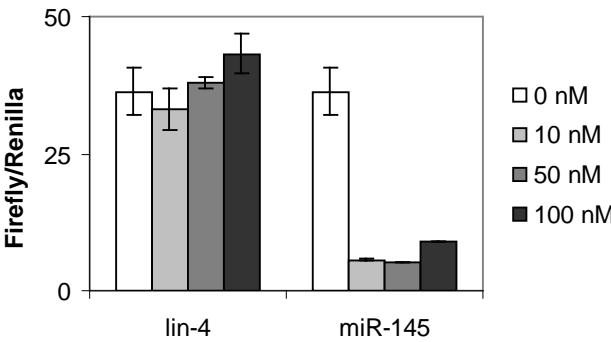

Supplement: Figure S2 — Validation of miR-145 overexpression. Firefly luciferase reporter containing a miR-145 (pMIR-145-REPORT) complementary site (perfect antisense sequence) was co-transfected with a Renilla luciferase transfection control plasmid and the indicated amounts of miRNA duplexes in DLD-1 cells (A) and HCT-116 cells (B). Transfection with lin-4 was used as a non-specific control. Luminescence was measured 24 hours post-transfection and the firefly luciferase activity was normalized to the activity of the co-transfected Renilla plasmid. Data are shown as the mean ± S.D. of four replicates. (0.01 MB PDF) [file pone.0008836.s002.pdf]

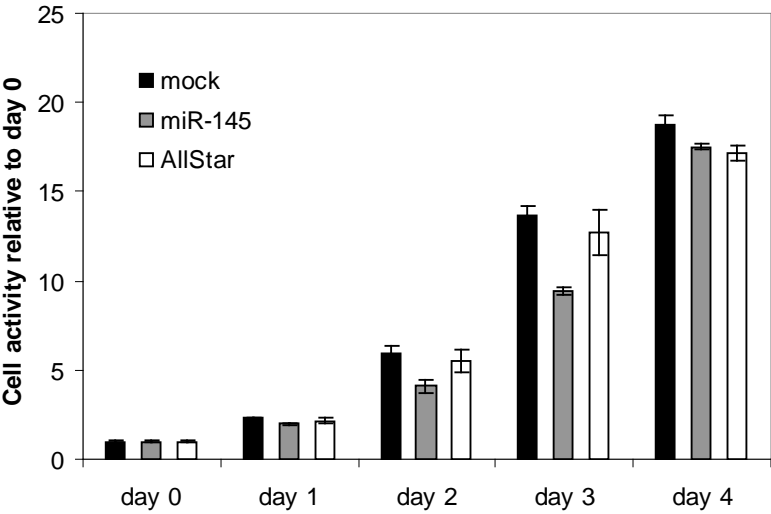

Supplement: Figure S3 — MTT cell proliferation assay. DLD-1 cell proliferation as measured by MTT assay upon transfection with 50 nM miR-145 duplex, 50 nM AllStar negative control or mock transfection. Data are shown as the mean ± S.D. of four replicates. (0.01 MB PDF) [file pone.0008836.s003.pdf]

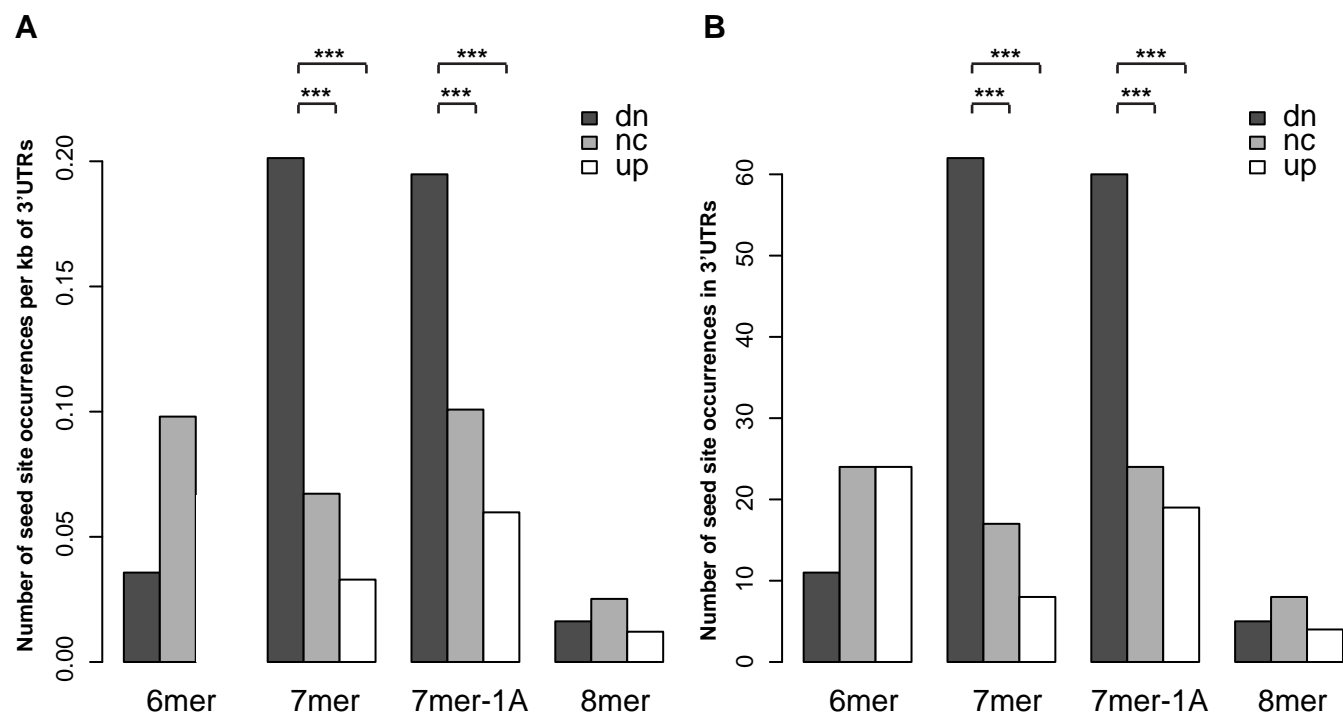

Supplement: Figure S4 — Seed site enrichment reported per kb and as counts. A, Seed site occurrences in the 3′UTRs of up, down and no-change transcripts for miR-145 presented per kb. The p-values were calculated as described in FIGURE 2. P-values for 7mer seed site enrichment were 2.3.10-28 (dn vs. up) and 5.1.10-7 (dn vs. nc). P-values for 7mer-1A seed site enrichment were 1.3.10-14 (dn vs. up) and 8.7.10-4 (dn vs. nc). B, Seed site occurrences in the 3′UTRs of up, down and no-change transcripts for miR-145 after correction of the up, down and no-change gene sets to the same size (scaling down the sets to the size of the smallest). The p-values were calculated as described in FIGURE 2. P-values for 7mer seed site enrichment were 3.7.10-13 (dn vs. up) and 1.7.10-8 (dn vs. nc). P-values for 7mer-1A seed site enrichment were 1.4.10-7 (dn vs. up) and 1.2.10-5 (dn vs. nc). (0.01 MB PDF) [file pone.0008836.s004.pdf]

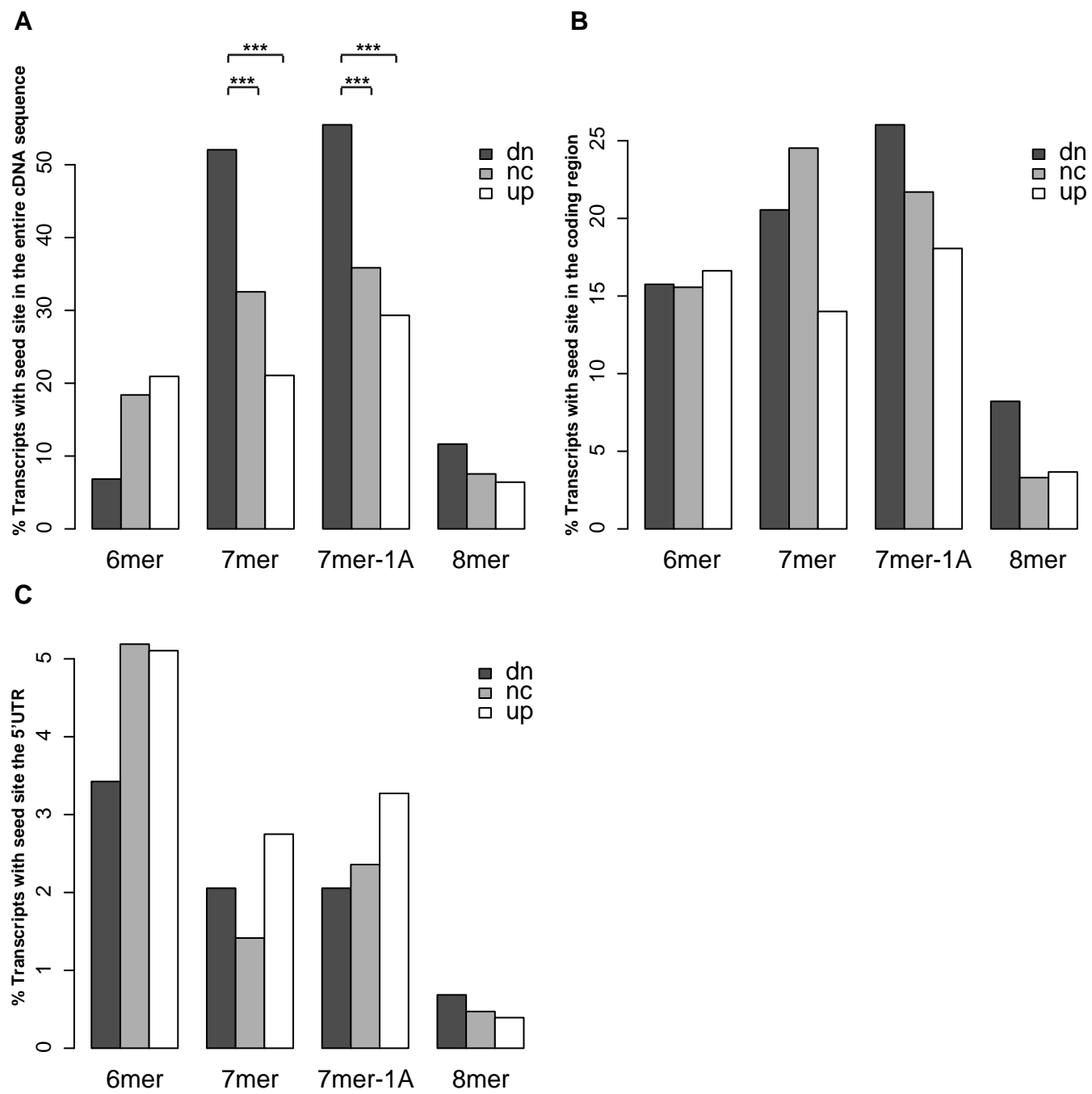

Supplement: Figure S5 — Seed site enrichment reported for cDNA sequences, coding regions and 5′UTRs. The percentage of genes in the up, down and no-change sets with seeds sites calculated for the entire cDNA sequences (A), coding regions (B) and 5′UTRs (C). P-values for 7mer seed site enrichment in cDNA sequences were 7.7.10-10 (dn vs. up) and 1.3.10-3 (dn vs. nc). P-values for 7mer-1A seed site enrichment were 6.7.10-6 (dn vs. up) and 1.5.10-3 (dn vs. nc). (0.01 MB PDF) [file pone.0008836.s005.pdf]

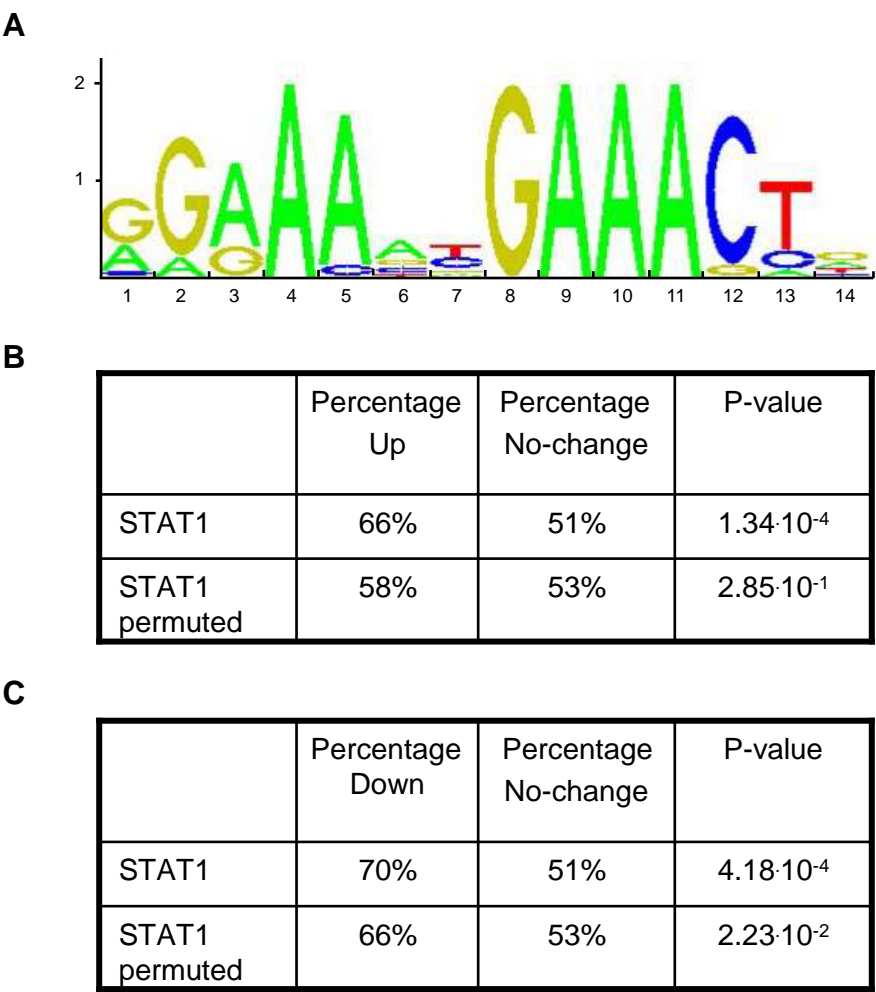

Supplement: Figure S7 — Enrichment of putative STAT1 binding sites in the promoters of up- and down-regulated genes. Binding motif for STAT1 as defined in the JASPAR database (A). Overrepresentation of STAT1 binding sites compared to a shuffled STAT1 binding site in promoters of down-regulated (B) and up-regulated (C) genes compared to promoters of genes with no change in expression level. (0.10 MB PDF) [file pone.0008836.s007.pdf]

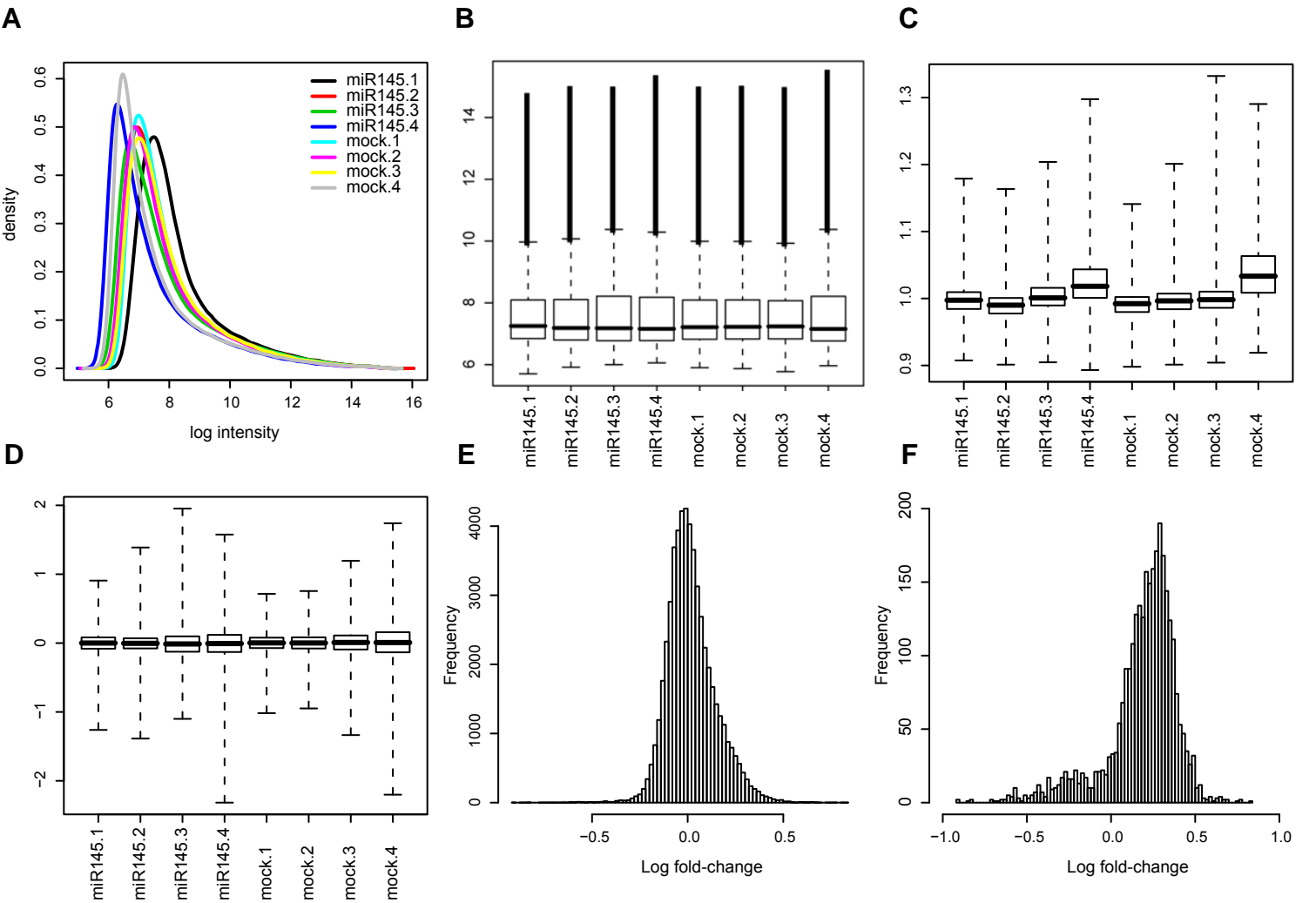

Supplement: Figure S8 — Microarray quality and processing. Histogram of raw log intensities for individual arrays before normalization (A). Boxplot of log intensities after normalization (B). The fit of the vsnrma model used to normalize the arrays were evaluated by calculation of normalized unscaled standard errors (C) together with relative log expression (RLE) plots showing the log expression for each probeset on each chip, relative to the median value for that probeset (D). Histogram of the log fold-change (logFC) distribution before (E) and after (F) non-specific filtering. (0.06 MB PDF) [file pone.0008836.s008.pdf]
