# Supplementary material for: MicroRNA-145 Targets YES and STAT1 in Colon Cancer Cells
Source: PLoS One. 2010 Jan 21;5(1):e8836. doi: 10.1371/journal.pone.0008836 (PMC2809101; doi:10.1371/journal.pone.0008836)
Supplement: Figure S6 — 6mer unbiased word analysis. A, Running sum of the overrepresentation score for the miR-145 6mer seed site in the ranked list of 3′UTR sequences (black line) compared to permutations of the ranked gene list (red lines). B, Top 10 enriched 6mer words in the 3′UTRs among the down-regulated transcripts. (0.41 MB PDF) [file pone.0008836.s006.pdf]

Gregersen et al. FIGURE S6

A

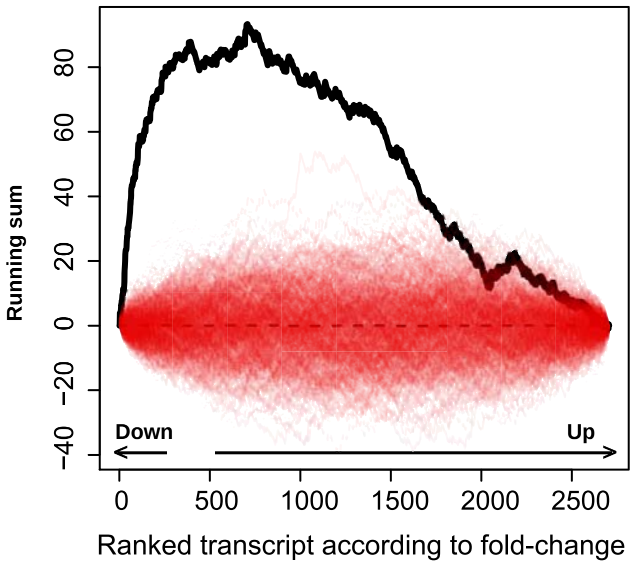

B

Rank	Word	z-Score	FDR	Annotation
1	**<u>AACTGG</u>**	7.16	<0.002	hsa-miR-145
2	T**<u>AACTG</u>**	6.12	<0.002	hsa-miR-935
3	ACTAGC	5.37	<0.002	
4	GTTAGC	5.20	<0.002	
5	**<u>ACTGGA</u>**	5.15	<0.004	hsa-miR-145
6	ATGCAT	4.92	<0.002	hsa-miR-586
7	ACTTTG	4.66	<0.006	hsa-miR-520g, hsa-miR-520h
8	TAAATG	4.45	<0.002	
9	CAGGAA	4.44	<0.002	
10	ATCATT	4.43	<0.002	
