# Supplementary material for: MicroRNA-145 Targets YES and STAT1 in Colon Cancer Cells
Source: PLoS One. 2010 Jan 21;5(1):e8836. doi: 10.1371/journal.pone.0008836 (PMC2809101; doi:10.1371/journal.pone.0008836)
Supplement: Table S1 — List of potential miR-145 targets (defined as downregulated transcripts with at least one miR-145 7mer, 7mer-1A or 8mer seed site in their 3′UTRs). (0.11 MB DOC) [file pone.0008836.s009.doc]

## Table S1: List of potential miR-145 targets (defined as downregulated transcripts with at least one miR-145 7mer, 7mer-1A or 8mer seed site in their 3’UTRs)

| **Ensembl Gene Identifier** | **HGNC Symbol** | **logFC** | **P-value** | **FDR** | **6mer** | **7mer** | **7mer-1A** | **8mer** |
| --- | --- | --- | --- | --- | --- | --- | --- | --- |
| ENSG00000175348 | TMEM9B | -0,905 | 2,7E-05 | 3,3E-02 | 2 | 2 | 2 | 0 |
| ENSG00000146386 | C6orf115 | -0,838 | 1,7E-04 | 5,7E-02 | 1 | 1 | 1 | 0 |
| ENSG00000133789 | SWAP70 | -0,833 | 7,9E-05 | 3,9E-02 | 3 | 1 | 2 | 0 |
| ENSG00000034713 | GABARAPL2 | -0,713 | 1,4E-04 | 5,7E-02 | 1 | 1 | 0 | 0 |
| ENSG00000149573 | MPZL2 | -0,702 | 1,4E-03 | 1,2E-01 | 4 | 2 | 2 | 0 |
| ENSG00000113583 | C5orf15 | -0,653 | 4,1E-04 | 8,1E-02 | 1 | 1 | 1 | 0 |
| ENSG00000072415 | MPP5 | -0,652 | 3,4E-04 | 8,1E-02 | 3 | 1 | 2 | 0 |
| ENSG00000158769 | F11R | -0,625 | 9,2E-04 | 1,2E-01 | 3 | 2 | 1 | 0 |
| ENSG00000197045 | GMFB | -0,618 | 5,5E-04 | 8,5E-02 | 4 | 1 | 2 | 0 |
| ENSG00000107566 | ERLIN1 | -0,617 | 8,7E-04 | 1,2E-01 | 2 | 1 | 1 | 0 |
| ENSG00000109189 | USP46 | -0,616 | 4,8E-04 | 8,5E-02 | 2 | 1 | 0 | 0 |
| ENSG00000102471 | NDFIP2 | -0,602 | 2,6E-03 | 1,2E-01 | 1 | 1 | 1 | 0 |
| ENSG00000124786 | SLC35B3 | -0,599 | 2,1E-03 | 1,2E-01 | 1 | 0 | 1 | 0 |
| ENSG00000144959 | NCEH1 | -0,584 | 6,9E-04 | 9,7E-02 | 2 | 1 | 1 | 0 |
| ENSG00000110330 | BIRC2 | -0,577 | 1,4E-03 | 1,2E-01 | 2 | 1 | 1 | 0 |
| ENSG00000136379 | FAM108C1 | -0,572 | 2,2E-03 | 1,2E-01 | 6 | 3 | 4 | 1 |
| ENSG00000177971 | IMP3 | -0,572 | 1,5E-03 | 1,2E-01 | 1 | 1 | 1 | 0 |
| ENSG00000107897 | ACBD5 | -0,569 | 2,4E-03 | 1,2E-01 | 5 | 2 | 1 | 0 |
| ENSG00000060749 | QSER1 | -0,565 | 1,1E-03 | 1,2E-01 | 2 | 2 | 1 | 0 |
| ENSG00000180329 | CCDC43 | -0,564 | 9,6E-04 | 1,2E-01 | 2 | 1 | 1 | 0 |
| ENSG00000125821 | DTD1 | -0,564 | 1,1E-03 | 1,2E-01 | 1 | 1 | 0 | 0 |
| ENSG00000119979 | FAM45A | -0,561 | 6,8E-03 | 1,3E-01 | 1 | 1 | 1 | 0 |
| ENSG00000119979 | FAM45B | -0,561 | 6,8E-03 | 1,3E-01 | 1 | 1 | 1 | 0 |
| ENSG00000176105 | YES1 | -0,545 | 7,1E-03 | 1,3E-01 | 1 | 1 | 1 | 0 |
| ENSG00000128567 | PODXL | -0,512 | 3,5E-03 | 1,2E-01 | 4 | 0 | 2 | 0 |
| ENSG00000162704 | ARPC5 | -0,512 | 2,1E-03 | 1,2E-01 | 1 | 1 | 0 | 1 |
| ENSG00000024862 | CCDC28A | -0,509 | 2,1E-03 | 1,2E-01 | 1 | 1 | 0 | 0 |
| ENSG00000151665 | PIGF | -0,506 | 3,1E-03 | 1,2E-01 | 2 | 1 | 1 | 0 |
| ENSG00000076248 | UNG | -0,503 | 1,5E-03 | 1,2E-01 | 1 | 0 | 1 | 0 |
| ENSG00000124532 | MRS2 | -0,498 | 2,9E-03 | 1,2E-01 | 1 | 1 | 0 | 0 |
| ENSG00000144746 | ARL6IP5 | -0,489 | 6,5E-03 | 1,3E-01 | 2 | 2 | 1 | 0 |
| ENSG00000090054 | SPTLC1 | -0,474 | 3,5E-03 | 1,2E-01 | 1 | 1 | 0 | 0 |
| ENSG00000145725 | HISPPD1 | -0,467 | 1,0E-02 | 1,4E-01 | 1 | 1 | 0 | 0 |
| ENSG00000138814 | PPP3CA | -0,461 | 3,7E-03 | 1,2E-01 | 3 | 1 | 2 | 0 |
| ENSG00000159322 | ADPGK | -0,457 | 6,1E-03 | 1,3E-01 | 2 | 1 | 1 | 0 |
| ENSG00000086232 | EIF2AK1 | -0,45 | 3,3E-02 | 1,7E-01 | 2 | 1 | 1 | 0 |
| ENSG00000164983 | TMEM65 | -0,449 | 4,6E-03 | 1,3E-01 | 1 | 1 | 1 | 0 |
| ENSG00000023909 | GCLM | -0,448 | 8,2E-03 | 1,4E-01 | 2 | 1 | 1 | 0 |
| ENSG00000106484 | MEST | -0,447 | 7,6E-03 | 1,3E-01 | 1 | 1 | 1 | 0 |
| ENSG00000158417 | EIF5B | -0,446 | 2,0E-02 | 1,5E-01 | 1 | 1 | 0 | 0 |
| ENSG00000115159 | GPD2 | -0,445 | 3,9E-03 | 1,2E-01 | 5 | 2 | 1 | 0 |
| ENSG00000173218 | VANGL1 | -0,437 | 3,4E-03 | 1,2E-01 | 1 | 1 | 1 | 0 |
| ENSG00000116679 | IVNS1ABP | -0,432 | 5,1E-03 | 1,3E-01 | 1 | 1 | 1 | 0 |
| ENSG00000159128 | IFNGR2 | -0,431 | 6,0E-03 | 1,3E-01 | 1 | 1 | 1 | 0 |
| ENSG00000166747 | AP1G1 | -0,427 | 5,2E-03 | 1,3E-01 | 4 | 3 | 2 | 0 |
| ENSG00000139163 | ETNK1 | -0,427 | 1,6E-02 | 1,5E-01 | 3 | 1 | 2 | 0 |
| ENSG00000173744 | AGFG1 | -0,425 | 5,0E-03 | 1,3E-01 | 3 | 1 | 3 | 0 |
| ENSG00000152749 | GPR180 | -0,417 | 2,5E-02 | 1,5E-01 | 3 | 1 | 1 | 0 |
| ENSG00000068650 | ATP11A | -0,409 | 1,8E-02 | 1,5E-01 | 5 | 2 | 1 | 1 |
| ENSG00000158528 | PPP1R9A | -0,407 | 1,3E-02 | 1,5E-01 | 5 | 1 | 2 | 1 |
| ENSG00000198818 | SFT2D1 | -0,403 | 1,4E-02 | 1,5E-01 | 1 | 1 | 0 | 0 |
| ENSG00000147419 | CCDC25 | -0,382 | 1,1E-02 | 1,4E-01 | 1 | 1 | 1 | 0 |
| ENSG00000113161 | HMGCR | -0,376 | 1,2E-02 | 1,5E-01 | 1 | 1 | 1 | 0 |
| ENSG00000163817 | SLC6A20 | -0,369 | 1,4E-02 | 1,5E-01 | 1 | 0 | 1 | 0 |
| ENSG00000164172 | MOCS2 | -0,365 | 1,7E-02 | 1,5E-01 | 3 | 1 | 2 | 0 |
| ENSG00000052723 | SIKE1 | -0,36 | 3,5E-02 | 1,7E-01 | 1 | 1 | 1 | 0 |
| ENSG00000169519 | METT5D1 | -0,359 | 1,5E-02 | 1,5E-01 | 2 | 0 | 1 | 0 |
| ENSG00000074416 | MGLL | -0,359 | 3,3E-02 | 1,7E-01 | 4 | 2 | 0 | 1 |
| ENSG00000117862 | TXNDC12 | -0,359 | 2,3E-02 | 1,5E-01 | 1 | 0 | 1 | 0 |
| ENSG00000112685 | EXOC2 | -0,347 | 1,6E-02 | 1,5E-01 | 2 | 1 | 2 | 0 |
| ENSG00000158042 | MRPL17 | -0,342 | 3,4E-02 | 1,7E-01 | 1 | 1 | 0 | 0 |
| ENSG00000075618 | FSCN1 | -0,341 | 1,6E-02 | 1,5E-01 | 4 | 3 | 4 | 0 |
| ENSG00000140905 | GCSH | -0,336 | 2,7E-02 | 1,6E-01 | 1 | 1 | 1 | 0 |
| ENSG00000109654 | TRIM2 | -0,321 | 2,3E-02 | 1,5E-01 | 5 | 3 | 5 | 0 |
| ENSG00000196937 | FAM3C | -0,314 | 3,1E-02 | 1,6E-01 | 2 | 2 | 1 | 0 |
| ENSG00000120742 | SERP1 | -0,31 | 3,7E-02 | 1,7E-01 | 1 | 1 | 1 | 0 |
| ENSG00000175066 | GK5 | -0,307 | 3,9E-02 | 1,8E-01 | 4 | 1 | 1 | 0 |
| ENSG00000169398 | PTK2 | -0,3 | 4,5E-02 | 1,9E-01 | 2 | 0 | 1 | 0 |
| ENSG00000116984 | MTR | -0,299 | 3,1E-02 | 1,6E-01 | 1 | 1 | 1 | 0 |
| ENSG00000112294 | ALDH5A1 | -0,298 | 4,0E-02 | 1,8E-01 | 1 | 1 | 0 | 0 |
| ENSG00000156675 | RAB11FIP1 | -0,287 | 3,2E-02 | 1,7E-01 | 3 | 0 | 1 | 0 |
| ENSG00000135913 | USP37 | -0,282 | 7,2E-02 | 2,4E-01 | 1 | 0 | 1 | 0 |
| ENSG00000178537 | SLC25A20 | -0,28 | 4,0E-02 | 1,8E-01 | 2 | 1 | 0 | 0 |
| ENSG00000186866 | POFUT2 | -0,273 | 5,3E-02 | 2,0E-01 | 1 | 0 | 1 | 0 |
| ENSG00000100503 | NIN | -0,268 | 4,2E-02 | 1,8E-01 | 2 | 1 | 1 | 0 |
| ENSG00000204524 | ZNF805 | -0,263 | 6,7E-02 | 2,3E-01 | 1 | 0 | 1 | 0 |
| ENSG00000151694 | ADAM17 | -0,249 | 7,5E-02 | 2,5E-01 | 1 | 1 | 1 | 0 |
| ENSG00000165495 | PKNOX2 | -0,23 | 7,4E-02 | 2,4E-01 | 1 | 1 | 0 | 0 |
|  |  |  |  |  |  |  |  |  |
