# Supplementary material for: MicroRNA-145 Targets YES and STAT1 in Colon Cancer Cells
Source: PLoS One. 2010 Jan 21;5(1):e8836. doi: 10.1371/journal.pone.0008836 (PMC2809101; doi:10.1371/journal.pone.0008836)
Supplement: Table S2 — Primer sequences used for quantitative RT-PCR. (0.03 MB DOC) [file pone.0008836.s010.doc]

**Table S2: Primer sequences used for quantitative RT-PCR**

| **Gene** |  | **Forward sequence** |  | **Reverse sequence** |
| --- | --- | --- | --- | --- |
| BIRC2 |  | 5’-AAATGCTGCGGCCAACATCTTC-3’ |  | 5’-TTCCAGTGACAGACCTGAAACATC-3’ |
| GABARAPL2 |  | 5’-TCACTGTGGCTCAGTTCATGTGG-3’ |  | 5’-AGCTGTCCCATAGTTAGGCTGGAC-3’ |
| HPRT |  | 5’- ACCCGGGAAAGCAACTGTTTG-3’ |  | 5’- TCACCAGGATAAGCCTCACCAG-3’ |
| IQGAP1 |  | 5’- TGGAACCGTGGACCCAAAGAAC-3’ |  | 5’- ACCTCCGCTGATTCCGAATATCCC-3’ |
| STAT1 |  | 5’-ATTACTCCAGGCCAAAGGAAGCAC-3’ |  | 5’- AGCAAGGCTGGCTTGAGGTTTG-3’ |
| TMEM9 |  | 5’-AGACGCCGCCAAGAATTTCGAG-3’ |  | 5’-GCATGGGCTCCACAACATGAAG-3’ |
| VANGL1 |  | 5’-AGTAAAGAAGCGGAAAGCAAGGC-3’ |  | 5’-CTGGAGACGCTGAATGTGGATG-3’ |
| YES |  | 5’- TCCTGCTGGTTTAACAGGTGGTG-3’ |  | 5’- TGCTTCCCACCAATCTCCTTCC-3’ |
|  |  |  |  |  |
